# Comparison of the Transcriptomic Signatures of Skin and Lung Fibroblasts from Patients with Systemic Sclerosis

**DOI:** 10.70322/fibrosis.2026.10008

**Published:** 2026-05-15

**Authors:** Samantha E. Kotz, Ludivine Renaud, Carol A. Feghali-Bostwick

**Affiliations:** Department of Medicine, Division of Rheumatology and Immunology, MUSC, Charleston, SC 29425, USA;

**Keywords:** Systemic sclerosis, Scleroderma, Fibroblasts, Skin, Lung, RNA sequencing, Meta-analysis, Transcriptome

## Abstract

Systemic Sclerosis (SSc) is a chronic autoimmune disease characterized by fibrosis in connective tissues. Fibroblasts are the effector cells of fibrosis since they contribute to the production of collagen and other extracellular matrix components. The goal of this study is to compare the transcriptomic profiles of primary human SSc skin and SSc lung fibroblasts. First, we conducted a meta-analysis of differentially expressed (DE) genes from two previously published differential analyses (SSc *vs*. normal) using skin and lung fibroblasts, observing 8.7% overlap in DE genes and 30% overlap in impacted pathways. Next, we characterized the signature of several genes of interest from the pro- and anti-fibrotic programs within the unique and overlap groups and explored overlapping drugs that are predicted to revert DE genes to “normal expression”. Finally, we identified 3760 DE genes between SSc lung and SSc skin fibroblasts, highlighting that fibroblasts in the disease state carry a tissue-specific signature that should be taken into consideration for therapeutic development. We also identified core genes that can serve as common targets for both skin and lung in SSc. To our knowledge, this is the first study to describe overlapping genes and pathways in primary human skin and lung fibroblasts from SSc patients.

## 1. Introduction

Systemic Sclerosis (SSc, also known as scleroderma) is a chronic autoimmune disease that primarily affects connective tissues and often results in fibrosis due to the overproduction of extracellular matrix (ECM) components [1]. SSc predominantly affects the skin and internal organs such as the heart, kidneys, and lungs. SSc is more common in females than males, yet disease progression tends to be more severe in males [2,3].

SSc is categorized into diffuse cutaneous SSc (dcSSc), limited cutaneous SSc (lcSSc), and SSc *sine* scleroderma [4]. DcSSc is characterized by proximal skin fibrosis that can involve the trunk, upper arms, thighs and face, and shows rapid disease progression and severe organ involvement early in the course of the disease [5,6]. In lcSSc, skin thickening is progressive and restricted to the distal limbs and the face [7]. Inclusion of samples from patients with lcSSc can provide valuable information on skin fibrosis progression despite the low modified Rodnan skin score (mRSS) characteristic of lcSSc [8]. In SSc *sine* scleroderma, patients have internal organ involvement but no skin thickening.

SSc-associated interstitial lung disease (SSc-ILD) is observed in both dcSSc and lcSSc patients and results in impaired function [9]. SSc-ILD is currently the leading cause of death in SSc patients [10]. Because disease progression is similar and progressive in the skin of lcSSc patients, irrespective of disease duration, and the lungs of SSc-ILD patients, this study will focus on comparing the transcriptomic signatures of lcSSc skin fibroblasts (SSc skin) and SSc-ILD lung fibroblasts (SSc lung).

Fibroblasts are responsible for the excess production of ECM that leads to fibrosis [11,12]. Fibroblasts are a valuable tool for understanding the mechanisms of fibrosis and for testing pro-fibrotic triggers and anti-fibrotic therapies. Fibroblasts can be cultured from both lung and skin tissues of healthy and diseased donors and retain the phenotype of the original tissue [13]. We conducted several studies to capture disease-associated transcriptional differences of SSc skin and SSc lung fibroblasts by comparing them to their respective healthy control counterparts [14,15], but we have not previously compared transcriptional differences of SSc skin and SSc lung fibroblasts. Thus, the goal of this study is to compare the transcriptomic signatures of SSc skin and lung fibroblasts to identify transcriptomic similarities and differences.

## 2. Materials and Methods

### 2.1. Selection of Datasets for SSc Skin and SSc Lung Fibroblasts

**Figure 1.**
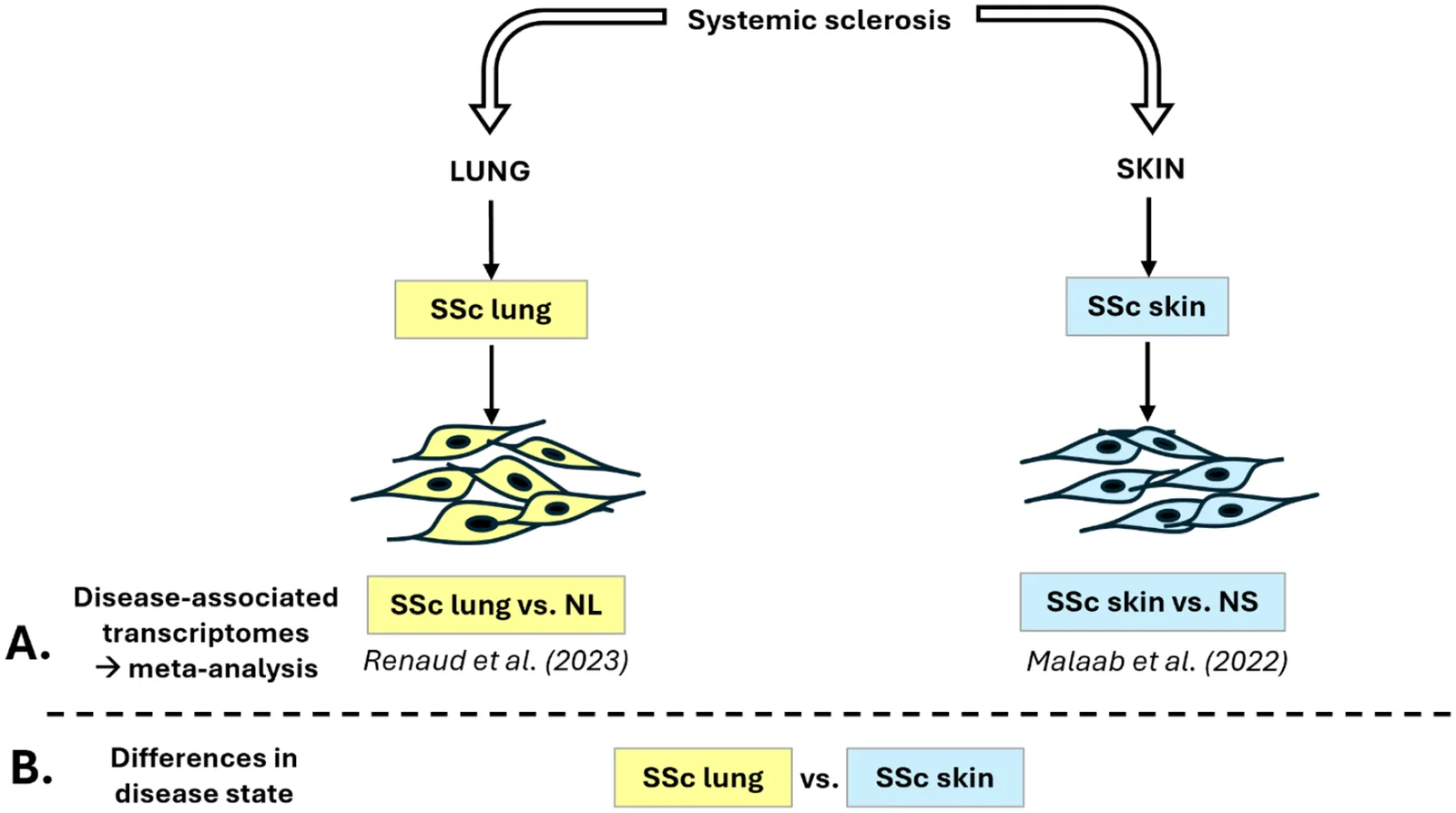
Experimental design. (**A**) Experimental design for the meta-analysis comparing disease-associated transcriptional differences in SSc lung and SSc skin fibroblasts. (**B**) Experimental design for the differential expression analysis “SSc lung *vs*. SSc skin” fibroblasts. NL: normal lung. NS: normal skin.

The differentially expressed (DE) genes used in this study were obtained from two of our published datasets (Figure 1A): (1) the SSc-ILD fibroblasts dataset (SSc lung) is from Renaud et al. [14], specifically from the European American cohort (NCBI GEO accession# GSE215841), and (2) the lcSSc skin fibroblasts dataset (SSc skin) is from Malaab et al. [15] (NCBI GEO accession# GSE153880).

### 2.2. Differential Expression Analysis & Meta-Analysis

The differential expression analysis “SSc *vs*. normal” was conducted on both skin and lung fibroblasts to determine disease-associated transcriptional differences using DESeq2 package [16] (Figure 1A). DE genes were determined based on 2 criteria: FDR adjusted *p*-value (*q*-value) < 0.1 and log2 fold change (log2FC) > |0.6|. The meta-analysis was performed on iPathwayGuide (Advaita), generating Venn diagrams for genes, pathways, and Gene Ontology terms, including Biological Process (BP), Molecular Function (MF), and Cellular Component (CC). The significance cutoff for pathways and Gene Ontology is using the *p*-value (*p*-value < 0.05).

A new differential expression analysis was conducted, “SSc lung *vs*. SSc skin” (*q*-value < 0.1, log2FC > |0.6|), to determine differences in disease state by comparing SSc lung and SSc skin fibroblasts with the same package and criteria of significance (Figure 1B). The same library preparations were performed by Novogene (NEBNext^®^ UltraTM RNA library prep kit for Illumina, NEB, Ipswich, MA, USA) on the two RNAseq datasets. To account for possible batch effect, selected DE genes were validated by qPCR using the same RNA used for RNA sequencing.

### 2.3. cDNA and qPCR Validation

A NanoDrop Lite spectrophotometer (ThermoFisher Scientific, Waltham, MA, USA) was used to quantify RNA concentration and quality. To synthesize cDNA, 1 μg of RNA per 20 μL was used with random hexamers and the SuperScript IV reverse transcriptase (Invitrogen, Carlsbad, CA, USA) on a C1000 Touch Thermal Cycler (Bio-Rad, Hercules, CA, USA). Quantitative PCR (qPCR) was performed to measure mRNA expression levels using the TaqMan real-time PCR system (Life Technologies, Carlsbad, CA, USA) according to the manufacturer’s protocol on a TaqMan Gene Expression Assays Step One Plus real-time PCR system (Life Technologies) using the following TaqMan human primers: *ACTA2* (Hs00426835_g1), *COL3A1* (Hs00943809_m1), *CTHRC1* (Hs00298917_m1), *IL6* (Hs00985639_m1), and *LOX* (Hs00184700_m1). The housekeeping genes *GAPDH* (FAM Hs02758991_g1, abbreviated *GAP*) and *B2M* (VIC Hs00187842_m1) were duplexed. The target gene 2^−ΔCT^ values normalized to *GAP* or *B2M* were statistically analyzed in GraphPad Prism version 10.4.1 (GraphPad Software Inc., La Jolla, CA, USA).

### 2.4. Heatmaps

A list of genes of interest (GOI) was generated with genes that are known to be markers of alveolar or inflammatory fibroblasts, pro-fibrotic genes, ECM genes, or anti-fibrotic genes (Supplemental Table S1). This list of GOI was then merged with the DE genes that are (1) unique to SSc-ILD fibroblasts, (2) unique to lcSSc fibroblasts, or (3) overlapping. A second “merge” was performed to obtain the counts from the original differential expression analysis. Morpheus was then used to generate heatmaps (https://software.broadinstitute.org/morpheus/, accessed on 1 February 2026) using the “Kmeans clustering” method (metric: one minus Pearson correlation; clustering first on columns, then on rows; maximum iterations: 1000). For the overlapping DE genes, since 2 sets of counts were available for SSc-ILD pulmonary and lcSSc dermal fibroblasts, 2 heatmaps were generated.

### 2.5. Statistical Analysis

GraphPad Prism 10 version 10.4.1 (GraphPad Software, Inc.) was used for statistical analysis. To identify outliers, the ROUT method was used. A two-tailed unpaired *t*-test with 95% confidence level was performed on the qPCR validation dataset. *p* < 0.05 was considered significant: * *p* < 0.05, ** *p* < 0.01, *** *p* < 0.001, **** *p* < 0.0001. Error bars = SEM.

## 3. Results

### 3.1. Meta-Analysis: Disease-Associated Transcriptomes of SSc Skin and SSc Lung Fibroblasts

**Figure 2.**
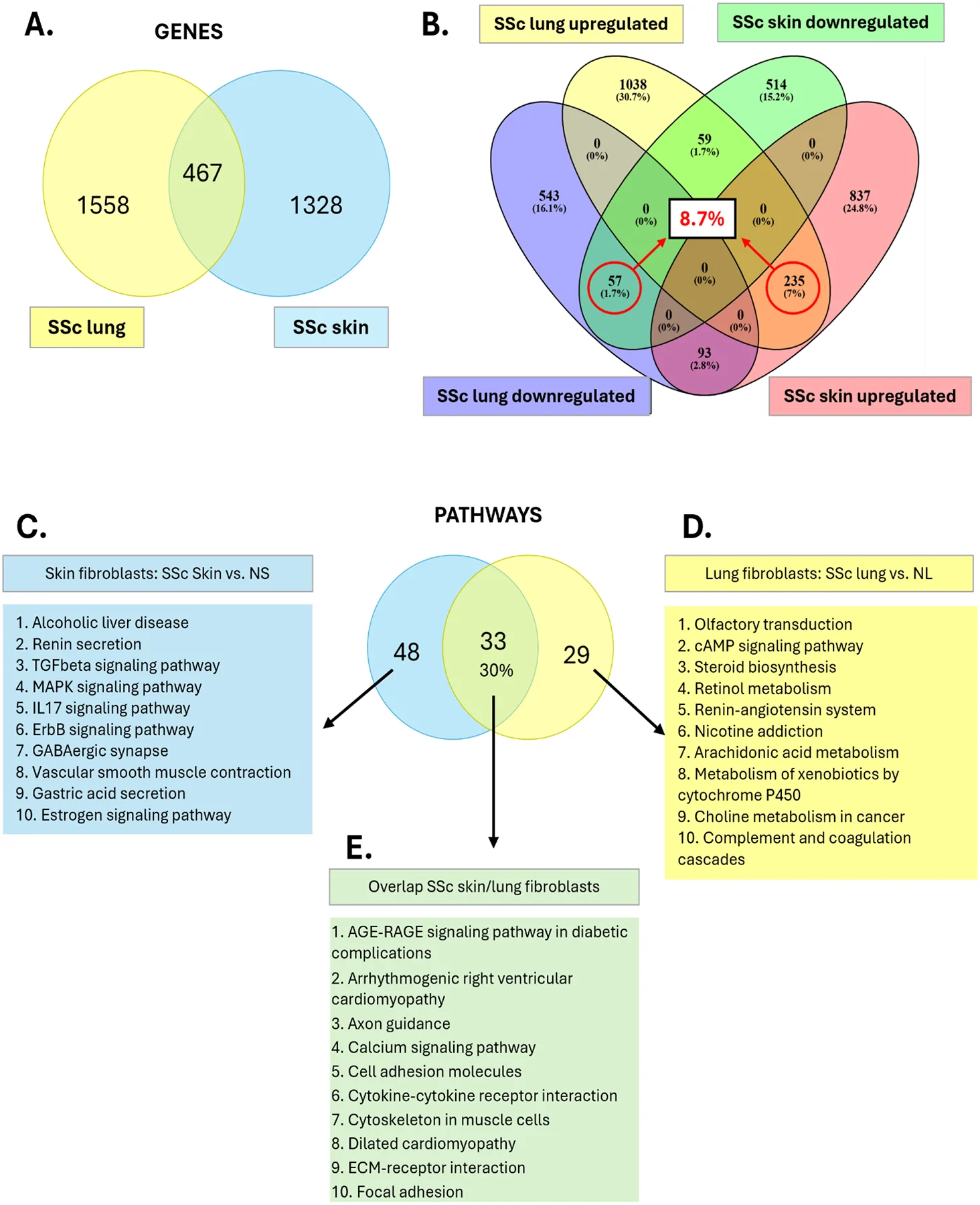
Meta-analysis: genes and pathways. (**A**) Venn diagram (2 entries) of genes unique or overlapping in SSc lung and SSc skin fibroblasts. (**B**) Venn diagram (4 entries) of upregulated or downregulated genes in SSc lung and SSc skin fibroblasts. (**C**) Top 10 impacted pathways unique to SSc skin fibroblasts. (**D**) Top 10 impacted pathways unique to SSc lung fibroblasts. (**E**) Top 10 impacted pathways overlapping between SSc skin and SSc lung fibroblasts.

We previously performed the differential expression analysis “SSc *vs*. normal” in (1) lung fibroblasts of SSc-ILD patients (SSc lung) versus normal lung (NL) fibroblasts from healthy control donors, and (2) skin fibroblasts of lcSSc patients (SSc skin) versus site-matched normal skin (NS) of healthy control donors (Figure 1A). The number of DE genes for each differential expression analysis is shown in Table 1. The meta-analysis shows that 467 DE genes overlap between SSc skin and SSc lung while 1328 are unique to SSc skin fibroblasts and 1558 are unique to SSc lung fibroblasts (Figure 2A). Out of the 467 overlapping DE genes between SSc skin and SSc lung fibroblasts, 235 are commonly upregulated while 57 are commonly downregulated, representing 8.7% of the total DE genes (Figure 2B, Supplemental Table S2). Note also that amongst the 467 overlapping DE genes, 59 are upregulated in SSc lung while downregulated in SSc skin fibroblasts, and 93 DE genes are downregulated in SSc lung while upregulated in SSc skin fibroblasts, emphasizing that “overlapping DE genes” can also have opposite regulation depending on the tissue of origin.

### 3.2. Meta-Analysis: Impacted Pathways in SSc Skin and SSc Lung Fibroblasts

Focusing on impacted pathways, the meta-analysis (Supplemental Table S3) revealed that: (1) 48 pathways are unique to SSc skin fibroblasts (Figure 2C), including “Alcoholic liver disease”, “Renin secretion”, “TGFβ signaling pathway”, “MAPK signaling pathway”, “IL17 signaling pathway”, “ErbB signaling pathway”, “GABAergic synapse”, “Vascular smooth muscle contraction”, “Gastric acid secretion”, and “Estrogen signaling pathway”, pointing to a biological state characterized by intense immune and inflammatory activation, rewired cell signaling, and metabolic and mitochondrial reprogramming, all of which suggest a highly stressed or disease-altered cellular environment. (2) 29 pathways are unique to SSc lung fibroblasts (Figure 2D), including “Olfactory transduction”, “cAMP signaling pathway”, “Steroid biosynthesis”, “Retinol metabolism”, “Renin-angiotensin system”, “Nicotine addiction”, “Arachidonic acid metabolism”, “Metabolism of xenobiotics by cytochrome P450”, “Choline metabolism in cancer”, and “Complement and coagulation cascades”, highlighting metabolic reprogramming (especially lipid and steroid metabolism), activation of detoxification/xenobiotic pathways, cancer-associated pathways and hormone-driven signaling, suggesting a system under chemical stress with strong endocrine and metabolic adaptation. (3) 33 pathways overlapped between SSc skin and SSc lung fibroblasts, representing 30% of all pathways (Figure 2E). Among these, the top 10 most impacted were “AGE-RAGE signaling pathway in diabetic complications”, “Arrhythmogenic right ventricular cardiomyopathy”, “Axon Guidance”, “Calcium signaling pathway”, “Cell adhesion molecules”, “Cytokine-cytokine receptor interaction”, “Cytoskeleton in muscle cells”, “Dilated cardiomyopathy”, “ECM-receptor interaction”, and “Focal adhesion”, reflecting a broad remodeling of core cell-signaling networks, altered cell–cell/matrix communication, and disease-associated and immune-linked pathways, pointing to a system undergoing structural and regulatory remodeling.

### 3.3. Balance Between Pro and Anti-Fibrotic Programs in SSc Skin and SSc Lung Fibroblasts

**Figure 3.**
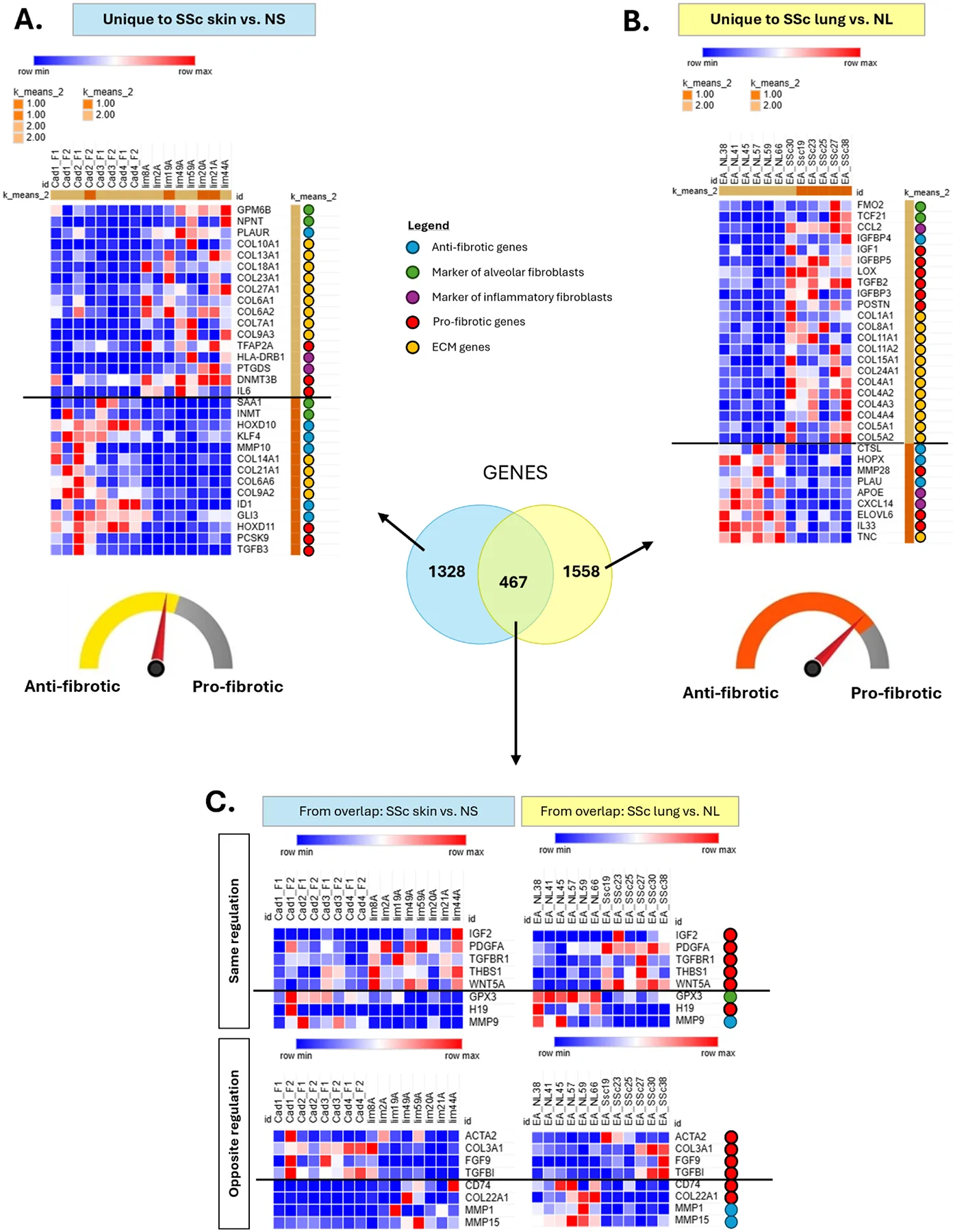
Signature of GOI in SSc skin and SSc lung fibroblasts. (**A**) Heatmap of GOI in SSc skin fibroblasts. (**B**) Heatmap of GOI in SSc lung fibroblasts. (**C**) Heatmaps of GOI in the overlap between SSc skin and SSc lung fibroblasts.

To focus on the deregulation of genes of interest (GOI, Supplemental Table S1) implicated in fibrosis and inflammation collated from the scientific literature, we generated heatmaps specific to the DE genes that are unique to SSc skin fibroblasts and SSc lung fibroblasts (Figure 3). In SSc skin fibroblasts (Figure 3A), many ECM genes, markers of inflammatory fibroblasts and pro-fibrotic genes are upregulated as expected, including *COL10A1*, *COL13A1*, *COL18A1*, *COL23A1*, *COLL27A1*, *COL6A1*, *COL6A2*, *COL7A1*, *COL9A3*, *TFAP2A*, *HLA-DRB1*, *PTGDS*, *DNMT3B* and *IL6*, but several ECM and pro-fibrotic genes are also downregulated, such as *COL14A1*, *COL21A1*, *COL6A6*, *COL9A2*, *HOXD11*, *PCSK9* and *TGFB3*. Additionally, markers of alveolar fibroblasts and anti-fibrotic genes were upregulated (*GPM6B*, *NPNT*, *PLAUR*) while others were downregulated (*SAA1*, *INMT*, *HOXD10*, *KLF4*, *MMP10*, *ID1*, *GLI3*) in SSc skin fibroblasts, suggesting that the balance between the pro- and anti-fibrotic programs is likely balanced in these fibroblasts, but inflammation and immune responses are active.

In SSc lung fibroblasts (Figure 3B), most of the markers of inflammatory fibroblasts, ECM, and pro-fibrotic genes from our list of GOI are upregulated, including *CCL2*, *IGF1*, *IGFBP5*, *LOX*, *TGFB2*, *IGFBP3*, *POSTN*, *COL1A1*, *COL8A1*, *COL11A1*, *COL11A2*, *COL15A1*, *COL24A1*, *COL4A1*, *COL4A2*, *COL4A3*, *COL4A4*, *COL5A1* and *COL5A2*. Only *MMP28*, *APOE*, *CXCL14*, *ELOVL6*, *IL33* and *TNC* are downregulated genes from the pro-fibrotic and inflammatory program in these fibroblasts. Additionally, the anti-fibrotic genes *CTSL*, *HOPX* and *PLAU* are downregulated in SSc lung fibroblasts, except for *IGFBP4*, which is upregulated, along with the markers of alveolar fibroblasts *FMO2* and *TCF21*. Together, these results emphasize that the balance is tipped in favor of the pro-fibrotic program in the disease-associated transcriptional signature of SSc lung fibroblasts than it is in the SSc skin fibroblast signature, with both the IGF and TGFβ signaling pathways represented.

As shown above, among the 467 overlapping DE genes between SSc skin and SSc lung fibroblasts, only 292 had the same direction of regulation, representing 8.7% of the total DE genes (Figure 2B). In Figure 3C we show that, included in these 292 DE genes, are (i) the upregulated pro-fibrotic genes *IGF2*, *PDGFA*, *TGFBR1*, *THBS1* and *WNT5A*, and (ii) the downregulated anti-fibrotic gene *MMP9* along with the marker of alveolar fibroblasts *GPX3* and the long non-coding RNA *H19*. Also present in the overlap are pro-fibrotic genes with opposite regulation in SSc skin and SSc lung fibroblasts, including (iii) *ACTA2*, *COL3A1*, *FGF9* and *TGFBI* that are downregulated in SSc skin fibroblasts while upregulated in SSc lung fibroblasts, and (iv) *CD74* and *COL22A1* that are upregulated in SSc skin fibroblasts while downregulated in SSc lung fibroblasts. Note also that the anti-fibrotic genes *MMP1* and *MMP15* are upregulated in SSc skin fibroblasts while downregulated in SSc lung fibroblasts.

### 3.4. Predicted Drugs in the Overlap Between SSc Skin and SSc Lung Fibroblasts

**Figure 4.**
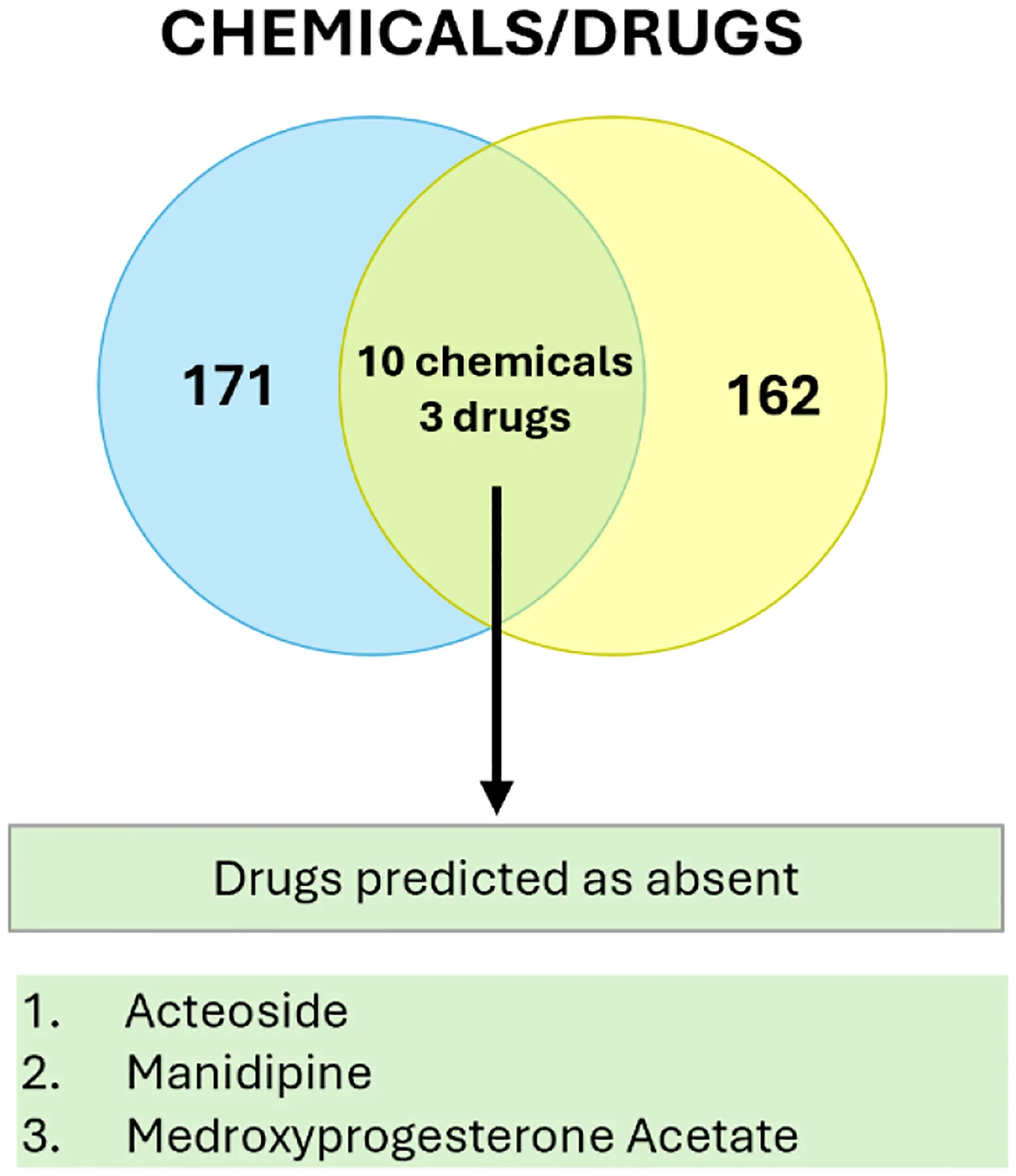
Meta-analysis—Upstream Regulators, Chemicals & Drugs. Drugs overlapping between SSc skin and SSc lung fibroblasts.

The iPathwayGuide “upstream regulators” tool returned 10 chemicals out of which 3 are classified as “drugs” predicted as absent/insufficient (Figure 4). These drugs, if introduced in the cells, are predicted to bring levels of expression closer to the phenotypic signatures of non-fibrotic fibroblasts: the bioactive natural product “acteoside”, the calcium channel blocker “manidipine”, and “medroxyprogesterone acetate”. The small overlap in drugs between SSc skin and SSc lung fibroblasts reinforces the differentiation between the two subtypes and highlights the need for tissue-specific therapeutic development.

### 3.5. Differences in Disease State: “SSc Lung vs. SSc Skin” Fibroblasts

**Figure 5.**
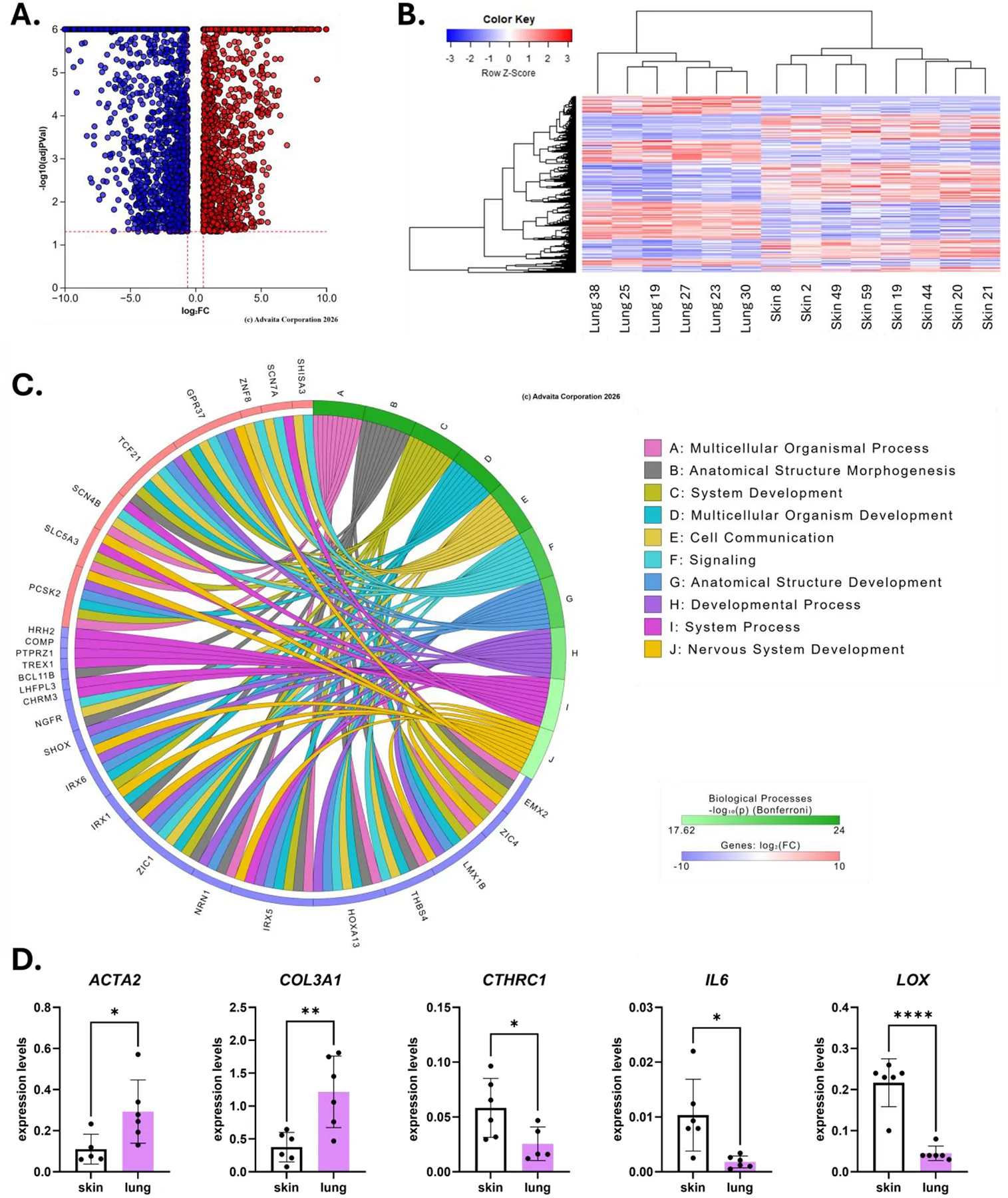
Differences in disease state: “SSc lung *vs*. SSc skin” fibroblasts. (**A**) Volcano plot for the differential expression analysis “SSc lung *vs*. SSc skin” fibroblasts (*q*-value < 0.1, log2FC > |0.6|). (**B**) Heatmap generated by DEseq2 for all genes with a *q*-value < 0.1. (**C**) Ribbon plot generated by iPathwayGuide for the top 10 enriched pathways showing the top 10 DE genes contributing to each pathway. For panels (**B**–**D**): downregulated DE genes are shown in blue, upregulated DE genes are shown in red. (**D**) Validation of selected DE genes in SSc skin and SSc lung fibroblasts by qPCR. * P < 0.05, ** P < 0.01, **** P < 0.0001 relative to skin.

To compare the SSc phenotype of lung and skin fibroblasts, we performed a new differential expression analysis using the raw counts of SSc lung and SSc skin fibroblasts from the 2 differential expression analyses previously published and used for the meta-analysis described above. The differential expression analysis “SSc lung *vs*. SSc skin” fibroblasts returned 3760 DE genes, out of which 1724 are upregulated and 2036 are downregulated in SSc lung compared to SSc skin fibroblasts (Figure 5A,B, Supplemental Table S4). This gene signature impacted 95 pathways (Figure 5C, Supplemental Table S5), including “Cell adhesion molecules”, “ECM-receptor interaction”, “Viral protein interaction with cytokine and cytokine receptor”, “Focal adhesion”, “Neuroactive ligand-receptor interaction”, and “Regulation of actin cytoskeleton”. Selected DE genes were validated by qPCR (Figure 5D) and confirmed the upregulation of *ACTA2* and *COL3A1* mRNA levels and the downregulation of *CTHRC1*, *IL6* and *LOX* in SSc lung fibroblasts relative to SSc skin fibroblasts.

## 4. Discussion

The goal of this study was to compare the transcriptomic signatures of SSc skin and SSc lung fibroblasts. First, our meta-analysis identified 292 overlapping DE genes between SSc skin and SSc lung fibroblasts, representing 8.7% overlap, and 33 (30%) commonly impacted pathways. From the signature of selected GOI, we defined that SSc lung fibroblasts seem to be in a more active state of fibrosis, while inflammatory activation is more pronounced in SSc skin fibroblasts. We also explored drugs predicted to restore the overlapping DE genes to “normal expression” when introduced to the fibroblasts. Finally, we identified 3760 DE genes between SSc lung fibroblasts and SSc skin fibroblasts, highlighting that fibroblasts in SSc disease state carry a tissue-specific signature that should be taken into consideration for therapeutic development.

### 4.1. Meta-Analysis: Overlapping DE Genes and Pathways

Several studies have explored targets that impact fibrosis in either SSc skin or SSc lung fibroblasts, but to our knowledge, this study is the first meta-analysis to report disease-associated genes and pathways that are either unique or overlapping between SSc skin and SSc lung fibroblasts. At the gene level, our data show minimal overlap, only 8.7% (292 DE genes), between SSc skin and SS lung fibroblasts. However, the impacted pathway analysis revealed a 30% overlap. This emphasizes how difficult it would be to predict lung involvement based on SSc skin transcriptome or to identify therapies that would be equally effective in skin and lung fibroblasts. However, within the 30% overlapping pathways, several fundamental pathways are present and are discussed below.

The most enriched pathway in the overlap is the “AGE-RAGE signaling pathway in diabetic complications”, known to elicit activation of multiple intracellular signal pathways involving NADPH oxidase, protein kinase C, and MAPKs, leading to NFkB activity that promotes the expression of pro-inflammatory cytokines such as IL1, IL6, and TNFα. Moreover, AGE-RAGE interaction induces JAK-STAT and PI3K-Akt dependent pathways that regulate cell proliferation and apoptosis, respectively, as well as hypoxia-mediated induction of EGR1.

The “Calcium signaling pathway” is also in the overlap between SSc skin and SSc lung fibroblasts. This pathway is a major contributor to fibrosis as calcium oscillations activate fibroblast differentiation into myofibroblasts, increase ECM production and collagen secretion, and activate fibrotic signaling [17]. Blocking calcium signals as a therapeutic strategy to prevent fibrosis has been considered in dermal, pulmonary, and cardiac fibroblasts [18–21].

Cell adhesion is a central driver of SSc-related fibrosis as it controls how fibroblasts interact with their environment and neighboring cells, and how they respond to stiffening of the ECM [22]. The “Cell adhesion molecules” pathway is enriched in both SSc skin and SSc lung fibroblasts in our meta-analysis. These molecules are (glyco)proteins expressed on the cell surface, including integrins, immunoglobulins, selectins, and cadherins, and play a critical role in a wide array of biologic processes, including hemostasis, the immune response, inflammation, embryogenesis, and the development of neuronal tissue [23]. Another axis of communication between cells and their environment is the “Cytokine-cytokine receptor interaction” pathway, also enriched in the overlap. Cytokines can activate innate and adaptive inflammatory host defenses, cell growth and death, differentiation, angiogenesis, and developmental and repair processes aimed at restoring homeostasis. TGFβ and several cytokines are implicated in SSc [24,25] as they activate fibroblasts, amplify ECM production, and sustain a chronic inflammatory–fibrotic loop involving Th2 cells, growth-factor pathways, and altered fibroblast signaling [26]. Additionally, the “ECM-receptor interaction” pathway contributes to control of cellular activities such as adhesion, migration, differentiation, proliferation, and apoptosis by allowing interactions between cells and the ECM via transmembrane molecules.

Our meta-analysis revealed that once skin and lung fibroblasts have reached SSc disease state, they show little gene and pathway overlap, highlighting that fibroblasts take on a particular molecular phenotype depending on the tissue of origin. It has been shown that adult fibroblasts maintain the embryonic gene signature of their organ of origin, explaining fibroblast heterogeneity across different homeostatic tissues [27]. We show here that this specific phenotype remains distinct even in disease states, reinforcing the need for organ-specific strategies for targeted control of fibrosis.

### 4.2. A Gene Signature of Active Fibrosis in SSc Lung Fibroblasts

The heatmaps generated for well-known factors involved in fibrosis and all DE collagens showed that SSc lung fibroblasts are likely in a more active state of inflammation and fibrosis compared to SSc skin fibroblasts, as shown by the upregulation of several pro-inflammatory and pro-fibrotic factors. Noticeably, several factors belonging to the IGF signaling pathway are expressed at higher levels in SSc lung fibroblasts; *IGF1*, which is known for stimulating fibroblasts differentiation into myofibroblasts [28], and *IGFBP3*/*IGFPB5*/*LOX*, which have been described as central mediators of fibrosis by contributing to ECM deposition and inducing fibroblast migration [29–32]. Other pro-fibrotic pathways are also represented, including the TGFβ pathway via upregulation of *TGFB2* [33,34]. Unlike *TGFB1*, which is significantly upregulated in both IPF and SSc lung fibroblasts, *TGFB2* is only upregulated in SSc lung fibroblasts in our analysis, and is induced by another pro-fibrotic growth factor—*IGF2* [34]. Note that *IGF2* and *TGFBR1* are both overlapping upregulated genes in skin and lung fibroblasts, but the upregulation of *TGFB2* is only observed in SSc lung fibroblasts, and surprisingly, *TGFB3* is uniquely downregulated in SSc skin compared to lung fibroblasts in our dataset. This is not consistent with a previous report [35], however, our analysis uaws dermal fibroblasts from lcSSc patients of different disease duration while most studies use dermal fibroblasts from early disease dcSSc patients [36]. This could also explain why we identified *POSTN* as exclusively upregulated in SSc lung fibroblasts, whereas most of the literature reports it as upregulated in SSc skin fibroblasts [37,38]. Taken together, our findings show that the IGF and TGFβ signaling pathways are active in SSc lung fibroblasts.

MMP28, the most recently identified MMP, has pro-fibrotic properties. MMP28-deficient mice are protected from bleomycin-induced lung fibrosis, and overexpression of MMP28 accelerated epithelial cell proliferation, protected them against apoptosis, enhanced cell migration, and decreased E-cadherin expression while increasing fibronectin [39]. Another unique characteristic of MMP28 is that it can be found in both the cytoplasm and the nucleus of lung epithelial cells. We show here that this pro-fibrotic MMP is uniquely downregulated in SSc lung fibroblasts as compared to healthy controls.

We also captured the downregulation of many anti-fibrotic genes in SSc lung fibroblasts, including *CTSL*, *PLAU*, and *HOPX*. CTSL is considered an anti-fibrotic proteinase because it is necessary for the cleavage of collagen type XVIII to release endostatin, a potent anti-fibrotic protein that regulates ECM proteins, LOX, and EGR1 [40–42]. PLAU is another ECM-remodeling proteinase that promotes fibrosis resolution and activates MMPs, and we have previously shown that PLAU (uPA) and its receptor PLAUR (uPAR) mediate the anti-fibrotic effects of an anti-fibrotic peptide derived from endostatin [43]. Downregulation of *HOPX* has also been reported in IPF alveolar epithelial cells and may contribute to the loss of regenerative processes in end-stage IPF lungs [44]. The overall fingerprint we observed in SSc lung fibroblasts suggests that anti-fibrotic molecules are downregulated while several pro-fibrotic factors are upregulated, indicating that the fibrosis is progressive despite the severity of disease in explanted SSc lungs.

### 4.3. A Signature of Persistent Inflammation in SSc Skin Fibroblasts

In SSc skin fibroblasts, the upregulation of *IL6*, *HLA-DRB1*, and *PTGDS* [25,45–47], together with the downregulation of *SAA1* [48], indicates a shift toward an IL6-predominant, antigen-presenting inflammatory state, with reduced acute-phase signaling and enhanced prostaglandin D2-linked modulation. Increased expression of *PLAUR* [43] and the markers of alveolar fibroblasts, *NPNT* and *GPM6B* [49], combined with reduced *MMP10*, suggest reorganization of the uPA/uPAR pericellular niche. This combination would favor stable cell–matrix anchoring by limiting uPA driven plasmin generation and downstream MMP activation, thereby constraining matrix degradation and suppressing the acquisition of an invasive fibroblast phenotype [43,50,51]. Developmental and pro-fibrotic transcriptional programs are also altered as shown by the downregulation of pro-fibrotic factors (*TGFB3*, *HOXD11*) [52], and anti-fibrotic factors (*ID1*, *GLI3*, *HOXD10*, *KLF4*) [15,53–55], alongside the upregulation of pro-fibrotic genes (*TFAP2A*, *DNMT3B*) [15,56] suggesting attenuation of canonical TGFβ, EMT, Hedgehog/GLI, and HOX pathways, despite ongoing epigenetic remodeling. Finally, decreased *PCSK9* and *INMT*, together with increased *PTGDS*, point to shifts in lipid and metabolic signaling that may modulate inflammatory and remodeling responses. Collectively, the signature of SSc skin fibroblasts is characterized by persistent inflammation and matrix engagement, but reduced TGFβ/EMT developmental signaling compared to lung fibroblasts, indicating a distinct remodeling phenotype within the lcSSc skin microenvironment.

### 4.4. The Overlapping Signature of SSc Skin and SSc Lung Fibroblasts

Among our selected GOI (Supplemental Table S1), only 8 showed deregulation in the same direction in SSc skin and lung fibroblasts. Of these, several growth factors were increased in SSc in both skin and lung fibroblasts, including *PDGFA*, *WNT5A*, *FGF9*, and *IGF2*. Interestingly, *PDGFA* has been shown to increase TGFβ1 levels in human lung fibroblasts [57], and *IGF2* is increased in SSc lung fibroblasts [58]. *IGF2* increases collagen type I, collagen type III, and fibronectin production in fibroblasts [59], and the fact that its expression is upregulated in both SSc skin and lung fibroblasts suggests that *IGF2*, similarly to *PDGFA* and *WNT5A*, is a therapeutic target for both skin and lung in SSc patients. Most genes that show general involvement in inflammation and extracellular matrix regulation share similar directionality trends in both SSc skin and SSc lung fibroblasts, *i.e*., downregulation of *MMP9*, and upregulation of *THBS1* and *WNT5A*, suggesting that, similar to *PDGFA*, *WNT5A*, *FGF9*, and *IGF2*, these are core genes that should be considered when developing therapies to target both skin and lung fibrosis.

### 4.5. Different Collagens Are Deregulated in SSc Skin and SSc Lung Fibroblasts

We examined the profile of collagens, given that 44 collagen genes which encode 28 collagen proteins exist in humans [60]. We consulted ColPTMScape, a collagen post-translational modification (PTM) database managed by the Proteomics Lab that offers “human” and “lung” queries, and the enrichment of the well-known *COL1A1* along with *COL4A1*, *COL4A2*, *COL5A1* and *COL5A2* was reported in human lungs. In our results, we identified 12 collagen genes uniquely upregulated in SSc lung fibroblasts, including *COL1A1*, 4 isoforms of collagen type IV (*COL4A1*, *COL4A2*, *COL4A3*, *COL4A4*), 2 isoforms of collagen type V (*COL5A1*, *COL5A2*), and 2 isoforms of collagen type XI (*COL11A1*, *COL11A2*). Collagen type IV is a major component of the basement membrane that is poorly studied in the context of pulmonary fibrosis [45]. Collagen type V is also found in basement membranes, where it plays a role in cell adhesion and matrix repair, and it can polymerize with collagen type I in lungs to create heterotypic fibrils [46,47]. Others have reported increased expression of collagen type V in early skin disease in SSc patients [48], but in our study focused on lcSSc skin fibroblasts, we did not capture that signature. Collagen type V and XI are structurally and functionally closely related and can form rare hybrid collagen molecules [49]. Interestingly, *COL11A1* and *COL11A2* are also exclusively upregulated in SSc lung fibroblasts. Together, they seem to play a fundamental role in fibrillogenesis by creating a core buried within major collagen fibrils, yet they can still regulate cell adhesion and wound healing [49].

The SSc skin fibroblast heatmap shows 14 DE collagen genes, including multiple members of the collagen type VI and type IX families. The signature of DE genes from the pro- and anti-fibrotic programs is more equally distributed between the upregulated and downregulated groups. For example, from the collagen type VI family of non-fibrillar collagens that are implicated in ECM organization and regulation of dermal matrix assembly, composition, and fibroblast behavior [47], we report upregulation of *COL6A1* and *COL6A2*, accompanied by the downregulation of *COL6A6*, in SSc skin fibroblasts. Interestingly, *COL6A6* silencing resulted in an increase in MMP1 [48], which could infer anti-fibrotic properties, an unexpected feature for a collagen type VI.

A noticeable trend in SSc skin fibroblasts is the split of the “fibril-associated collagens with interrupted triple helices” (FACIT) that includes types IX, XII, and XIV. Collagens in this subclass share unique structural characteristics such as short triple-helical domains, multiple interruptions in the triple helix, large non-collagenous domains, and flexible hinge regions [49]. They do not form fibrils, but instead bind, stabilize, and regulate the fibrils made by other collagens, and are crucial for the stability of the ECM. Since the creation of this subclass, new collagens have been added, including COL21A1 [50]. Here we report that *COL9A3* and *COL22A1* are upregulated in SSc skin fibroblasts, while *COL9A2*, *COL12A1*, and *COL14A1* are downregulated. We previously reported increased expression of *COL22A1* in SSc dermal fibroblasts and induction of its expression by TGFβ in skin in organ culture [61]. Note that in SSc lung fibroblasts, only one FACIT, *COL22A1*, was reported as downregulated as compared to healthy controls. We also observed the upregulation of two collagens that have anti-fibrotic properties in SSc skin fibroblasts: *COL7A1* and *COL18A1*. As mentioned earlier, cleavage of COL18A1 by CTSL produces the anti-fibrotic matrikine endostatin [43–45]. The COL7A1 gene encodes anchoring fibrils, which are essential for the stability of the skin and other epithelial organs, and genetic loss results in dystrophic epidermolysis bullosa with chronic skin fragility and fibrosis [51,52].

### 4.6. Drugs Predicted to Return SSc Skin and SSc Lung Fibroblasts to “Normal State”

Leveraging our meta-analysis, 292 overlapping DE genes and two candidate drugs were identified as potential upstream regulators in SSc skin and SSc lung fibroblasts. Acteoside is a natural phenylethanoid glycoside from plants that has anti-inflammatory, anti-oxidant, and anti-tumor properties and is reported to treat cardiovascular disease, diabetes, and cancer [62,63]. In both murine and human lung fibroblasts, acteoside (aka verbascoside) inhibited TGFβ1-induced collagen type I expression via downregulation of Smad/non-Smad pathway and oxidative stress [64]. It also delayed the progression of renal interstitial fibrosis in diabetic nephropathy by anti-oxidation and regulating the autophagy-lysosome pathway [65].

Manidipine, a 3rd generation dihydropyridine calcium channel blocker used to treat mild to moderate hypertension, is also a predicted drug in the overlap of our meta-analysis. In fibrosis-related studies, manidipine (a) inhibited the expression of fibrillar collagens type I, type III, type IV, and the metallopeptidase inhibitor 2 TIMP2, (b) boosted the expression of matrix metallopeptidases 2 and 7 (MMP2, MMP7), and (c) regulated autophagy and inflammation, contributing to the reduction in renal fibrosis [66,67]. In mesangial cells, manidipine inhibited the transcription of several cytokines, including interleukin 1β (IL1β), but it also increased IL6, a known pro-fibrotic factor overexpressed in SSc [25,68]. Taken together, the upstream regulators analysis led to the identification of two potential drugs of interest that could have a beneficial impact on both skin and lung fibrosis, neither one of these drugs has been tested in patients with SSc or related fibrosing diseases.

### 4.7. SSc Lung vs. SSc Skin Fibroblasts: Differences in Disease State

More than 3500 DE genes were identified in “SSc lung *vs*. SSc skin” analysis, highlighting the considerable phenotypic differences in disease state in these fibroblasts. We validated that mRNA levels of *ACTA2* and *COL3A1* are higher in SSc lung fibroblasts, while levels of *CTHRC1*, *IL6*, and *LOX* are lower compared to SSc skin fibroblasts. Amongst the top 10 impacted pathways in SSc lung fibroblasts compared to SSc skin fibroblasts are the fundamental pathways “Cell adhesion molecules”, “ECM-receptor interaction”, “Focal adhesion” and “Cytokine-cytokine receptor interaction”. These were also reported in the meta-analysis that examined DE genes between healthy and diseased fibroblasts, but here we show profound differences in diseased fibroblasts originating from SSc-ILD lungs and lcSSc skin. As mentioned earlier, the fibroblast phenotype in homeostatic or disease state reflects the embryonic gene signature of the organ of origin [27], but we should also consider disease status as another factor generating differences, as SSc-ILD lungs were from severe pulmonary fibrosis, while lcSSc skin fibroblasts were collected from patients with different disease duration.

### 4.8. Limitations

The study has several limitations. Fibroblasts were from patients of varying disease duration, treatment history, and ages, which may all impact gene expression. In addition, lung fibroblasts are from explanted lungs, which reflect severe end-stage disease and thus might not reflect earlier disease stages. Lastly, skin and lung fibroblasts were from different donors.

## 5. Conclusions

To our knowledge, this is the first study comparing gene expression and pathways in human primary skin and lung fibroblasts from SSc patients. Our meta-analysis revealed that the overlap in DE genes, including collagens, and pathways is small, highlighting how different the phenotypic signatures of fibroblasts are when derived from different organs, and the need to take these signatures into consideration when developing therapeutic strategies to reverse fibrosis. SSc skin fibroblasts are dominated by immune and cytokine-driven activation, with strong inflammatory and pro-fibrotic signaling, as well as mitochondrial and metabolic stress. In contrast, SSc lung fibroblasts exhibit activation of the IGF and TGFβ signaling pathways, deep metabolic rewiring, particularly in lipid and steroid pathways, together with strong xenobiotic detoxification and hormone-linked/cancer-like signaling. These differences in skin and lung fibroblast signatures suggest that currently available treatments might not be equally effective in both tissues. From the overlap, we captured that SSc skin and lung fibroblasts share a conserved pathogenic signature defined by rewired core signaling pathways, altered cell–matrix communication, and immune-driven transcriptional dysregulation. The overlapping pathways reveal that, despite tissue-specific pressures, SSc fibroblasts converge on a common activated, stress-adapted, and pro-fibrotic state that underlies the systemic nature of the disease. Further, genes common to skin and lung fibroblasts, including *WNT5A*, *PDGFA*, and *IGF2*, can serve as common targets for the treatment of both skin and lung fibrosis in SSc.

## Supplementary Material

supplemental files

The following supporting information can be found at: www.sciepublish.com/xxx/s1, Table S1: Genes of interest; Table S2: Meta-analysis DE genes; Table S3: Meta-analysis pathways; Table S4: DE genes in SSc lung *vs*. SSc skin fibroblasts; Table S5: Pathways in SSc lung *vs*. SSc skin fibroblasts.

## Figures and Tables

**Table 1.** Summary of the DE genes returned by the differential expression analysis previously performed.

Comparison	# of DE Genes
SSc lung *vs*. NL	2025
SSc skin *vs*. NS	1795

## Data Availability

Data is available from the corresponding authors upon reasonable request. The RNAseq data used in this study have been deposited in the NCBI Gene Expression Omnibus (GEO) under accession numbers GSE215841 and GSE153880.
